# A Practical Way to Improve Dental College Instruction

**Published:** 1905-10

**Authors:** William H. Trueman

**Affiliations:** Philadelphia


### A PRACTICAL WAY TO IMPROVE DENTAL COLLEGE INSTRUCTION.

BY DR. WILLIAM H. TRUEMAN, PHILADELPHIA.

"At the Princeton Commencement President Wilson announced the new departure in instruction which he has been planning for some time past to introduce. A committee of the alumni has assured the University of additional income exceeding one hundred thousand dollars a year. This money is to be spent in adding to the Princeton faculty fifty preceptors, who are to do, apparently, what tutors do in the older English universities,—that is, they will keep in constant touch with the students, 'as guides, advisors, and testers of their learning.' Less reliance than formerly is to be placed at Princeton on recitations and examinations, and more on conferences of individuals and small groups of men with their instructors. Not only the new preceptors, but the older members of the faculty, are to take part in these conferences." (*Harper's Weekly*, July 1, 1905, page 931.)

This new departure is in line with the change that has been slowly going on in educational institutions. The instructor is being brought closer to the pupil. "Telling how," is giving way to the much more practical "showing how;" the gulf between the desk and the benches is slowly being bridged, and the printed text-book will soon be supplanted by well-trained, properly qualified demonstrators, who will interpret to individual students the instruction formally imparted from the lecture-table. This will call for a large increase in the teaching staff, a higher intelligence, and a marked aptness for teaching that will demand and merit a more liberal recompense. It will also make needful more room and more appliances. All these things are costly. The fees received from students do not, in the higher educational institutions, provide the facilities required.

While this fact has made an impress upon the community that has borne fruit, it does not seem to be appreciated by members of the dental profession. Judging from remarks now and again recorded as having been made at dental gatherings, the impression prevails that dental colleges are mines of wealth, and a dental professorship is a short cut to fortune. This is far, very far from being the case. While the cost of instructing has very much increased, the college fees have not been materially advanced, so that, notwithstanding the larger classes, that portion of the income charged to profit, from which the teaching staff is paid, is far below a just recompense for service rendered. The dental graduate seems impressed with the idea that his diploma is a *quid pro quo* for the fees he has paid; with its reception the transaction is ended; he has no further use for the college, and its future welfare is no concern of his.

It is very fortunate that this feeling does not extend to other vocations, otherwise educational facilities would be very meagre in this country. The medical fraternity contributes very largely to maintain the varied educational facilities of the medical profession. Its members give freely of their time, talent, and earnings, to found, maintain, and improve their colleges, hospitals, and libraries; and their example has prompted the laity to liberally assist. Their unselfishness is appreciated by the community, and in return the profession is freely accorded a social distinction and a place of honor as public benefactors.

The dental profession does not receive, and has done nothing to merit, any further consideration from the public than that accorded other artisans. The dentist does his work and gets his fees as a *quid pro quo*. While it is generally recognized by the profession that dental college training could readily be made more effective, any suggestion of providing the means is coldly received. A far less sum than that provided by the alumni of Princeton would enable any one of the dental colleges to vastly improve its teaching facilities, would attract to its teaching staff men of talent and ability who under present conditions forsake the schools for private practice, and the mere fact that if it had been done would raise the profession in public esteem. The excuse that the profession is poor is false. It is not dollars and cents, it is *esprit de corps* that is wanting. It is high time the dental profession fell in line, and in a practical way contributed to improve its own educational facilities. Let the faculties be assured that they will be financially supported in advancing the educational standard, and they will as readily respond as have other educational institutions. Loudly proclaiming that we are not commercialists, but professional men is idle talk so long as there is nothing publicly seen to show the existence of the dental profession but its business signs. We do nothing for the community but that which we are paid for, and for this we are constantly scheming for higher fees. The example of the Princeton alumni is an excellent one to follow, and the method of instruction liberally provided is just what is wanted to make the dental college course what it should be.

